# Powering cancer screening for overall mortality

**DOI:** 10.3332/ecancer.2013.ed27

**Published:** 2013-10-09

**Authors:** Vinay Prasad

**Affiliations:** Medical Oncology Branch, National Cancer Institute, National Institutes of Health, Bethesda, Maryland, USA

The goal of screening is to improve overall survival and quality of life, and yet, many of the campaigns currently recommended in the US lack robust, randomized controlled trial (RCT) evidence. As screening involves healthy patients, the demonstration that any potential intervention is beneficial is not merely scientific, but ethically imperative [1].

Historically, we have made assumptions. If a screening test for cancer decreases death from the target disease, and there is no obvious signal that any other type of death is increased, we have assumed that overall mortality (OM) is improved. In other words, the heuristic goes, if the trial’s power were larger, we would see this benefit. This reasoning buttresses nearly all widely used screening tests, but remains unproven in all but the rarest cases. Neither mammography, colonoscopy, sigmoidoscopy, fecal occult blood testing, prostate specific antigen screening, nor ultrasonography for abdominal aortic aneurysm (AAA) have shown overall mortality benefit in randomized controlled trials.

In the Prostate, Lung, Colorectal and Ovarian (PLCO) Cancer Screening Trial, screening with five modalities—combined—showed no benefit with respect to overall mortality. Only one, sigmoidoscopy, was ultimately found to be effective. In fact, to date, the only screening effort to show all causes of mortality benefit in RCT are spiral-computed tomography (CT) in the National Lung Screening Trial (NLST). CT screening may itself be a special case, as lung cancer is such a large driver of mortality in long-time, heavy smokers.

Unless overall mortality is directly improved by an intervention, we may always be unsure whether our fundamental assumption is correct. Prostate cancer screening may paradoxically increase death from competing causes, in this case, cardiovascular disease and suicide [2]. In most RCTs, such deaths may not be linked to the intervention—a problem called the slippery-linkage bias [3]. Could these deaths negate any tenuous gains made by screening?

Testing for overall mortality would require screening trials to be an order of magnitude larger than they are now. For instance the PLCO trial enrolled 155,000 participants, took more than 15 years to complete, and cost over 300 million dollars. To be adequately powered to assess all causes of death, a future trial may require 1 to 2 million participants, last over a decade, and cost upwards of a billion dollars. Such a costly research endeavor would almost surely be paid for by taxpayers, who will justifiably question whether their money is being wisely spent.

Spending this money is either wasteful or worth it, depending on the viewpoint you hold. From the point of view of federal agencies charged with dispersing a set pool of research funds, such as the National Institutes of Health, spending so much money on any single trial inherently comes at the price of forgoing many other pressing studies. One screening prevention mega-trial may cost as much as 50 well-done randomized trials for patients with metastatic cancer. From this viewpoint, it is hard to justify the expenditure.

However, from a 30,000-foot viewpoint of the federal government, such a study could be a good buy. Even at a price of 1 to 2 billion dollars, the costs of conducting such a study pale in comparison to the ongoing expenditures by the federal government on screening. From 2003 to 2008 alone, Medicare spent nearly 5 billion dollars [4] on cancer screening tests themselves. This figure does not include the costs of downstream diagnostic interventions, and treatment (including the overtreatment of some cases that would not cause harm, viz. “overdiagnosis”). For instance, in 2008, Medicare spent 1 billion dollars on intensity modulated radiation therapy for prostate cancer [4]. Cancer screening, and the cascade of events it prompts, costs taxpayers tens of billions of dollars each year. And, while it remains controversial whether diagnosing many cancers at an earlier state results in savings from metastatic treatment averted, it is certain that the extent of over diagnosis in cancer screening ensures that substantial spending is wasted [5]. Thus, only 1 to 2 % of the money spent on screening could test whether our efforts improve overall survival.

Moving towards a model of assessing overall mortality will require buy in from the public, professional community and elected officials. Patients should understand that without overall mortality benefit, doctors cannot say for certain that a test will help them live longer. While it remains important for patients to be presented with the risks and benefits of screening, lacking overall mortality data means that—to a large degree—the net benefits are not as certain as we would like them to be. And, if it were true that a prevention effort decreases death for some reason, but increases it equally for another, it should not be offered. For instance, cyclooxygenase-2 inhibitors suppress colon polyps, but worsen cardiovascular death. We cannot sweep dirt from the kitchen floor into the living room, and call it a clean house.

In concert with powering trials for overall mortality, future screening should not be disseminated until after clinical trials are conducted. Screening in the US has historically been at odds with this recommendation, yet the NLST suggests cautious adoption is achievable. Contamination with Chest CT of the control group occurred in 4.3% of patients in the NLST compared to 52% contamination by PSA (by year 6) in the PLCO study.

In the 1940s, when the first randomized trials were conducted, few would have imagined the mega trials of today, which have assessed all causes of mortality for complex treatment regiments and multicomponent intervention. Similarly, if buy-in for overall mortality reaches a critical mass, we may move towards a new standard of prevention trials. To get the most from the trials I propose, multimodalities could be assessed in a single study, as was done in the PLCO study. Alternatively, multinational collaborations may perform concurrent trials with standardized interventions and inclusion criteria. Collectively, the meta-study may be powered for OM, with each nation powered, at a minimum, for disease specific mortality. Such a trial design may balance the important issue of cost with the equally important issue of answering the question.

(Cancer) prevention and screening should be just that—a disease in parentheses. Because the goal of these efforts is one and the same, improving the quantity and quality of life, regardless of the reason for it ending.

The views expressed in this article are those of the author and do not necessarily reflect those of the National Cancer Institute or National Institutes of Health.
